# Treatment of Obstructive Sleep Apnea and Hipoapnea Syndrome with oral appliances

**DOI:** 10.1016/S1808-8694(15)31028-4

**Published:** 2015-10-19

**Authors:** Marco Antonio de Oliveira Almeida, Andressa Otranto de Britto Teixeira, Lívia Saladini Vieira, Cátia Cardoso Abdo Quintão

**Affiliations:** a PhD from the North Carolina University, Professor of Orthodontics at UERJ.; b Dental Surgeon, specialist in Orthodontics by ABO /JF, MSc Dentistry Student - Concentration Area: Orthodontics at UERJ; c Dental Surgeon, Orthodontics Specialization Student at UERJ; d PhD in Orthodontics at UFRJ, Associated Professor of Orthodontics at UERJ. State University of Rio de Janeiro.

**Keywords:** Apnea, Snoring, OSAHS, Oral Appliances

## Abstract

Obstructive sleep apnea and hipoapnea syndrome (OSAHS) is a disorder that affects about 4% of the adult population, and besides the social problems associated to snoring and extreme day time sleepiness, it is preoccupying since it may cause pulmonary hypertension and cardiac failure.

**Review and discussion:**

Through a literature review, we discuss the use of oral appliances to treat this condition, in regards of therapy effectiveness and limitations, main clinical symptoms, major occlusal side effects, rate of improvement and patient satisfaction.

**Conclusions:**

We concluded that the use of oral appliances should be a first choice treatment for mild to moderate OSAHS, being dental, joint and muscular discomforts, hypersalivation and xerostomia, the most frequent clinical symptoms, with light occlusal side effects that normally do not bother the patients, with a good degree of improvement and high satisfaction index.

## INTRODUCTION

Since 1985, Medicine and Dentistry have increased its focus on sleep respiratory disorders. Although there is a large number of disorders that may be included in this category, obstructive sleep apnea and the choices of its possible intra-oral treatments, have received more attention.

Obstructive sleep apnea and hypopnea syndrome (OSAH) is defined by the apnea/hypopnea index (AHI) per hour of at least five events per hour, added to clinical symptoms, being loud snoring and excessive daytime sleepiness, the most important ones.^1^, ^2^

This syndrome affects as many as 4% of the adult population, being more predominant in men, increasing from the 5th life decade onwards^3^, ^4^. Patients with OSAH have significantly more hypertension, ischemic heart disease and brain vascular disease than individuals without the syndrome. Mortality related to obstructive sleep apnea has increased significantly, when the AHI > 20 events per hour^5^.

Some important anatomic features observed radiographically in patients with OSAH include: narrow mandible arch; maxillary and mandibular retrognathism; increased lower facial height; lower and more anterior position of the hyoid bone; reduced pharyngeal area; increased cranio-cervical angle; decreased distance between the base of the tongue and the posterior pharyngeal wall; hypertrophied tonsils and adenoids^6^, ^7^, ^8^, ^9^, ^10^, over-erupted maxillary and mandibular dentition and enlarged tongue^6^, ^7^.

OSAH treatment goal is to restore normal breathing during sleep, and therefore, ending excessive daytime sleepiness, neuropsychological and cardiovascular alterations; and at the same time it should offer the patient good quality of life, without offering side effects or risks^11^.

Treatment options for OSAH include sleep hygiene, that is to say, avoidance of alcohol and other drugs; adequate body position and weight loss^11^, surgical procedures, such as partial glossectomy, uvulopalatopharyngoplasty, maxillary and mandibular advancement procedures, and also clinical treatments, such as nasal CPAP and intra-oral devices^12^.

The most commonly used clinical treatment includes the continuous positive air pressure procedure (CPAP) applied through a device that generates and directs a continuous air flow (40 to 60l/min.), through a flexible tube to a nasal mask tightly strapped to the patient's face.^3^, ^11^. According to Sullivan et al.^5^, although the treatment of obstructive sleep apnea with CPAP is highly effective, it is little tolerated by approximately 36% of patients, specially by those with medium severity.

There has been a recent increased interest on the principle of treatment with intra-oral devices, because they are a simple and non-invasive option, different devices have been developed.^5^. The devices approved by the Food and Drug Administration (FDA) are the tongue retainers and mandibular protruding devices, being the most widely used options^2^.

However, some questions need to be addressed in order to make this therapy into an efficient and safe alternative for treating such respiratory disorders. These questions are related to the correct indication regarding the severity of the condition^5^, that is to say, which degrees of apnea could be treated with this therapeutic option, patient's comfort when using the intra-oral device^13^, ^14^, ^15^, user's and room mate's evaluation regarding the improvement of the clinical condition^14^ and side effects.

The goal of this paper is based on the review of recent literature, and to check the following:

1 - Mandibular protruding devices efficacy for treating obstructive sleep apnea.
2 - Main clinical symptoms informed by patients.
3 - Main occlusal side effects produced by mandibular protruding devices.
4 - Patients’ collaboration level and satisfaction rate regarding the treatment.

## DISCUSSION

Regarding the efficacy of intra-oral devices, several authors state in their studies^16^, ^17^, ^18^, ^19^ that they represent a good alternative for treating snoring and OSAH, due to a reduced cost and their relatively comfortable use, being more accepted by patients. Although there are conflicting doubts on the efficacy of intra-oral devices for treating snoring and OSAH, its use in the treatment of medium severity apnea has received great attention and acceptability.^8^

A literature review done by Warunek (2004)10 showed that since 1985, approximately 150 articles have been published describing different devices and its efficacy in treating OSAH.

The mechanics of mandibular protruding devices aims to increase upper airway volume^2^, having as a result mandibular advancement, which places the tongue away from the posterior pharyngeal wall, lowering this bone and moving the tongue away from the soft palate^20^, altering the position of the hyoid bone and therefore, modifying the space of the hypopharyngeal airway.^3^

Regarding its effectiveness, Walker-Engström et al. (2002)^4^ managed to get at least a 50% improvement in the apnea - hypopnea index (AHI) in 81% of patients, and normalization in 63% of patients who used intra-oral devices which promoted mandibular advancement of 50% of protrusion maximum capacity. Using polysomnography, Neill et al. (2002)^14^ evaluated the use of intra-oral devices of mandibular protrusion and concluded that it was a complete success in 21.1% of patients, partial success in 52.6% and a failure in 26.3%, with an average treatment period of 6.5 weeks. According to the study done by Fritsch et al. (2001)^13^, a respiratory improvement was confirmed with the device, when all patients showed a persistent relief of symptoms, 12 to 30 weeks after its use.

In the study done by Pancer, Al-Faifi & Hoffstein (1999)20, they observed a significant reduction in all rates related to respiratory disorders and the subjective assessment of room mates revealed a striking improvement in snoring (96% were evaluated as “loud snoring” frequently before using the device and only 2% using the device). The objective analysis showed that of the 75 patients, 38 were cured (normalization of AH index), 31 improved AH index without normalization, three were only snorers, and three had a worse AH index with the device. According to Katto et al. (2000)^17^, among the patients who had mandibular advancement of at least 6mm, there was an improvement of their condition with normalization in 65%. They also reported that patients who normalized their indexes were the less obese of the study and suggested that obese patients should not be suitable candidates to therapy with oral devices. In the study done by Ferguson et al. (1997)^16^, when compared to the non-adjustable mandibular repositioning device, the adjustable mandibular protruding device, was associated with a higher success of treatment (55% vs. 48%), and fewer failures (5% vs. 24%). For Clark et al. (1993)^21^, the follow-up makes clear that when the protrusive position was lower than 75% of maximum protrusion reached by the patient (normally < 5mm) the advancement therapy did not work, and they stated that for those patients who do not tolerate such a degree of advancement, this would not be the indicated therapy.

For Fransson et al. (2004)^22^, the treatment with mandibular protruding devices has several advantages and can be considered as first choice treatment for a large group of patients, including patients with severe OSAH, if an optimum advancement quantity could be used. In our sample, more than 80% of patients with OSAH normalized their values of oxygen saturation (with an improvement of 50% or more), and 90% reported a subjective reduction of snoring and apnea (50% reduction or more).

Regarding clinical symptoms, dental or mouth discomfort were the most frequently symptoms reported by patients in all samples. In the work done by Neill et al. (2002)^14^, 26% of patients experienced some pain in the mouth and 42% reported sore teeth and gums. Pancer, Al-Faifi & Hoffstein (1999)^20^ reported dental discomfort some times or with frequency in more than 32% of patients. According to Clark, Sohn & Hong (2000)^3^, 38% of patients reported suffering from toothache. For O'sullivan (1995)^5^, the main effect reported was medium discomfort in the dental arcs when waking up, with reduction of the symptom in three weeks, in most cases. Besides discomfort, joint and masticatory musculature pain was also reported, found in different levels in the works by 2, 4, 8, 9, 12, 22. Other frequent clinical symptoms were excessive salivation^8^, ^13^ due to the new tongue position and to the presence of the device inside the mouth^2^, ^3^, ^8^, ^9^, ^12^, ^13^, ^14^, ^20^, and xerostomia^3^, ^5^. Fransson et al. (2004)^22^ and Bondemark & Lindman (2000)^9^ also reported the frequency reduction in headache in patients of their samples.

Among the frequently observed occlusal alterations, there are overjet, overbite reduction, lower incisive proclin ation^9^, ^10^, ^13^, ^18^, ^21^, ^22^, and the establishment of lateral open bite. 22 According to Fransson et al. (2002)^23^, a small overjet is a limiting factor to be considered in the treatment with this type of device.

According to Robertson (2001)^24^, the main changes observed are due to the vertical repositioning of the mandibular condyle in the glenoid cavity. These alterations may become visible within 6 months. Dental changes start happening later on, and they will be significant at the 30 month follow-up. Bondemark & Lindman (2000)^9^ carried out a two year follow-up work and they observed a significant decrease in overjet and overbite, and significant changes in molar relation, indicating a more mesial sagittal relation and/or changes in the incisive inclination.

Some authors^25^, ^26^, ^27^ correlate the observed occlusal changes with the type of initial occlusion in patients using mandibular protruding devices for OSAH treatment. Almeida et al. (2006)^25^ and Almeida et al. (2006)^26^ carried out some long term assessment works, 4.7 years average. Five orthodontists made a visual comparison of the dental arcades plaster molds of 70 patients, and 71 patients had their cephalometric x-rays analyzed. In the analysis of the models, the authors verified that changes occur in 85.7% of cases, being favorable or unfavorable depending on the initial mal-occlusion. On the other hand, on the cephalometric assessment, they observed a decrease of overjet and overbite, changes in the incisive and molar teeth position and consequent alteration in the mandibular position, also varying depending on the initial occlusion. Marklund (2006)^27^ confirmed this correlation between the observed changes and initial occlusion on their work with 187 patients, during an average follow-up period of 5.4 years.

The assessment of the patient compliance should be done in order to be able to indicate the treatment with mandibular protruding device in an effective manner, since this appears to be the main difficulty for treatment with CPAP. Compliance depends on the comfort level obtained by patient during the use of the devices and their own assessment regarding the result of the therapy. All the studies show a high level of compliance in using the device, and always in a higher level than the use of CPAP when they are compared^2^, ^3^, ^4^, ^5^, ^14^, ^16^, ^20^, ^22^. According to Almeida (2002)^2^ and Ferguson et al. (1997)^16^ patients have a significant preference for the use of mandibular protruding devices rather than the use of CPAP, even those for whom CPAP efficacy was better than intra-oral devices. Walker-Engströn et al. (2002)^4^ found a compliance level of 82% after one year and 62% after four years. Fransson et al. (2004)22 found an 84% compliance rate on the two year follow-up. Clark, Sohn & Hong (2000)^3^ reported that with the long term use (three or more years), compliance was higher than 51%, whereas Pancer, Al-Faifi & Hoffstein (1999)^20^ stated that 350 days after device fitting, 86% of patients still were using it. According to Neill et al. (2002)^14^ the device was used by 53% of patients every night, by 26% more than three night a week, by 21% less than that. A high satisfaction rate was reported by articles assessing this item.^4^, ^9^, ^20^.

## CONCLUSIONS

From the bibliography review focused on answering the main doubts on the use of intra-oral devices for treating OSAH, we can conclude the following:

1 - Intra-oral devices for mandibular advancement can be considered as first choice treatment for patients with medium to moderate OSAH, because the devices show a significant condition improvement and a greater acceptability from patients when compared to CPAP. These types of devices can be tried in patients with severe OSAH when they are intolerant to treatment with CPAP.
2 - The main clinical symptoms informed by patients are dental, joint and muscular discomfort, hyper salivation and xerostomia, reduction in headache frequency and improvement of snoring (frequency and intensity).
3 - The main occlusal side effects found were overjet reduction, overbite reduction, lower incisive proclination, establishment of lateral open bite. However, most of the time without causing great discomfort to patients.
4 - Treatment with mandibular protruding devices show a higher compliance level than

CPAP, even in patients who had a higher efficacy with CPAP than with intra-oral devices.

Regarding patient's satisfaction with treatment, it was observed a high rate in all the studies that included this item in their assessments.
